# Buffalo Medical and Surgical Association

**Published:** 1883-12

**Authors:** Clayton M. Daniels

**Affiliations:** Secretary


### Society Reports.

---

BUFFALO MEDICAL AND SURGICAL ASSOCIATION.

*Regular Meeting, November 6th, 1883.*

President, Dr. John Cronyn, in the Chair. Dr. C. M. Daniels, Secretary.

Present—Drs. Bartlett, Heath, Frank, Weil, Howe, Brecht, Putnam, Burghardt, Rochester, Coakley, Hartwig, Potter, C. A. Ring, and, by invitation, Dr. Keene.

Motion to suspend reading minutes of last meeting carried.

Application for membership received from Dr. James W. Hunter, who was duly elected.

The essayist, Dr. Howe, stated that he had been unable to complete his paper on "Blindness with Albuminuria," which he had intended to present, but would substitute the subject of "Artificial Illumination."

The paper referred in detail to the various means of artificial light used, from the tallow candle to that of electricity, and of their relative value, power, action on the eyes, etc., presenting many interesting points from the more recent investigations.

*Discussion*—Dr. Hartwig said he was sorry the essayist did not examine the oxy-hydrogen light, as he believed it would be the light of the future.

Dr. Heath expressed his appreciation of the paper, and referred to the inferior gas we were at times obliged to use. He thought no improvement would occur so long as the gas companies practically controlled the inspectorships.

He thought that the relative value of the electric light was in favor of the Swan and Sawyer storage batteries, because the Edison required the engine to go constantly. Also, that the cause of the irregularity of the Brush light was from faulty machinery.

Dr. Cronyn considered the subject an important one to students, especially those who "burn the midnight oil," as long continued use of improper light was exceedingly injurious.

Dr. Bartlett asked as to the best position light should be in for the benefit of the eyes.

Dr. Howe, in reply to Dr. Hartwig's reference to oxy-hydrogen as being the best light, said it was without doubt excellent when we are able to get it. Referring to the electric light "storage batteries," as mentioned by Dr. Heath, he thought they were not yet sufficiently perfected to be practical. In reply to Dr. Bartlett, said it should be behind or at left side.

*Voluntary Communications*—Dr. Hartwig asked for the experiences of members in opening abscesses after applying carbolic acid over point to be incised, as spoken of at the last meeting. He stated he had tried it several times with perfect success, not causing the least pain.

Dr. Heath said he had tried it by painting a line of carbolic acid over point of incision, but without diminishing the sensibility in the least; consequently he could not endorse it.

Dr. Hartwig replied that the success depended upon the manner of applying the acid. It required but very little; should be applied slowly with some sharp-pointed instrument, as a knife, and not spread out, as would be the case where a brush was used.

Dr. Rochester said it occurred to him that the tissues would not heal readily afterward and might leave a scar.

Dr. Hartwig remarked that it was usually desirable, when opening abscesses, that the wound remain open some considerable time. Also, that in his experience no scar was observable.

Dr. Cronyn thought ever so little carbolic acid might give a good deal of trouble by absorption, and that there would be danger in putting it on the knife. Referred to case where a glycerine and carbolic acid solution put in ear caused great renal irritation, albumen being present for a week.

Dr. Rochester thought not so much danger would be expected from strong solutions as from weak ones.

*Prevailing Diseases*—Scarlet fever, which seemed epidemic. Considerable diphtheria and some chicken pox. Dr. Rochester had noticed that when the public schools opened in the fall, scarlet fever became much more prevalent.

Dr. Bartlett said it seemed as if the school buildings might be a source of disease in holding the poison. Suggested proper fumigation.

Dr. Coakley had noted a peculiar eruptive disease resembling herpes, and chicken pox.

Adjourned. CLAYTON M. DANIELS, *Secretary.*

